# Tumor Cell Recognition Efficiency by T Cells

**DOI:** 10.1371/journal.pmed.0020077

**Published:** 2005-03-29

**Authors:** Daniel E Speiser, Jean-Charles Cerottini, Pedro Romero

**Affiliations:** Ludwig Institute for Cancer ResearchLausanneSwitzerland

Stuge et al. report a detailed analysis of the fine specificity of CD8+ T cells against tumor-associated antigen in melanoma patients [1]. They compared peptide-vaccination-driven with naturally arising T cell responses against the HLA-A*0201 restricted melanoma peptide antigens M26 (derived from Melan-A/MART-1) and G209-2M (derived from gp100 protein). A major endpoint of this study was in vitro tumor cell recognition by T cells. Fortunately, this is increasingly used as a “golden” standard in the assessment of tumor-specific T cells. The authors suggest that spontaneously arising antigen-specific T cell populations are qualitatively different from those induced by vaccination with heteroclitic peptides (which are altered for increased HLA binding): tumor cell recognition was found in nearly all T cells from the former, but only in a minority from the latter. As reported previously, these results correlated with recognition efficiency of antigenic peptides. We agree that this has considerable implications for immunotherapy and congratulate the authors for analyzing T cell recognition in great detail. However, in one point our own studies lead to different results: we repetitively found that the majority of T cells generated with the heteroclitic Melan-A M26 peptide were tumor reactive. This was the case for Melan-A-specific T cell populations generated in HLA-A*0201 transgenic mice [2], in vitro [3], and in melanoma patients [4]. The latter studies also assessed T cells from vaccination-site sentinel lymph nodes, containing T cells that are very likely selected and activated by vaccination and not by the tumor.

The authors point out correctly that tetramer+ T cells comprise many cells unable to recognize and kill tumor cells in an antigen-specific manner, presumably owing to low T cell receptor avidity to cognate antigen. An extreme case is naïve T cell populations, of which the majority are unable to recognize tumor cells, despite their specific binding to MHC/ peptide tetramers [5]. Therefore, it is crucial to exclude naïve T cells from studies analyzing tumor recognition. HLA-A*0201+ humans (healthy individuals and melanoma patients) have 0.07% ± 0.05% naïve Melan-A tetramer+ cells within peripheral blood CD8+ T cells [5,6]. The three patients studied by Stuge et al. had 0.23%, 0.12%, and 0.50% Melan-A tetramer+ cells. Thus, one can estimate that the studied populations from the three patients contained approximately 30%, 60%, and 15% naïve Melan-A-specific T cells, respectively. This is only a rough estimate—tetramer analysis before vaccination and assessment of CD45RA/ CCR7 expression would give more insight. Nevertheless, it remains likely that the first two patients had considerably more naïve cells than the third patient (i.e., the one without immunotherapy). In addition, naïve-derived CD8+ T cells have a higher clonogenic potential than activated Melan-A-specific T cells from melanoma patients (unpublished data). This means that overrepresentation of clones derived from naïve CD8+ T cells is likely to occur when both naïve and activated antigen-specific CD8+ T cells co-exist in a given lymphocyte population. As mentioned, Stuge et al. found unexpected high frequencies of T cell clones not recognizing tumor cells in the two vaccinated patients. It is conceivable that this was due to the presumably high percentages of naïve Melan-A-specific cells present in the populations used for generating the clones, which would provide an explanation for the discrepancy with the results of our studies [2,3,4].

Ethical considerations limit vaccination studies in healthy humans. In patients, candidate antigens should therefore be tested with strong adjuvants [7], to increase the likelihood that the studied responses are predominantly vaccination-driven, with only minor contribution of spontaneous T cell activation [8]. It would be desirable to directly compare vaccination with heteroclitic peptide versus vaccination with natural peptide. However, this is hampered by the lack of ex vivo detectable responses to native peptides owing to their low immunogenicity. Another option is to analyze clonal distributions (T cell receptors) of responding T cells extensively: Further support for the notion that spontaneous (tumor driven) responses have increased potential for tumor recognition would be obtained if mono/oligoclonal T cell repertoires are indeed significantly more often found in spontaneous than vaccination-induced responses.

We certainly agree that vaccines must be optimized. Thus, more such studies are desirable, since they have high potential to lead to better understanding of the differences between clinically irrelevant and relevant T cell responses, and to rapidly identify the most promising vaccine formulations that can subsequently be tested in large-scale clinical trials.
